# Identification of models of heterogeneous cell populations from population snapshot data

**DOI:** 10.1186/1471-2105-12-125

**Published:** 2011-04-28

**Authors:** Jan Hasenauer, Steffen Waldherr, Malgorzata Doszczak, Nicole Radde, Peter Scheurich, Frank Allgöwer

**Affiliations:** 1 Institute for Systems Theory and Automatic Control, University of Stuttgart, Germany; 2 Institute of Cell Biology and Immunology, University of Stuttgart, Germany

## Abstract

**Background:**

Most of the modeling performed in the area of systems biology aims at achieving a quantitative description of the intracellular pathways within a "typical cell". However, in many biologically important situations even clonal cell populations can show a heterogeneous response. These situations require study of cell-to-cell variability and the development of models for heterogeneous cell populations.

**Results:**

In this paper we consider cell populations in which the dynamics of every single cell is captured by a parameter dependent differential equation. Differences among cells are modeled by differences in parameters which are subject to a probability density. A novel Bayesian approach is presented to infer this probability density from population snapshot data, such as flow cytometric analysis, which do not provide single cell time series data. The presented approach can deal with sparse and noisy measurement data. Furthermore, it is appealing from an application point of view as in contrast to other methods the uncertainty of the resulting parameter distribution can directly be assessed.

**Conclusions:**

The proposed method is evaluated using artificial experimental data from a model of the tumor necrosis factor signaling network. We demonstrate that the methods are computationally efficient and yield good estimation result even for sparse data sets.

## Background

The main goals of research in systems biology are the development of quantitative models of intracellular pathways and the development of tools to support the modeling process. Thereby, most of the available methods and models consider only a single "typical cell" whereas most experimental data used to calibrate the models are obtained using cell population experiments, e.g. western blotting. This yields problems in particular if the studied population shows a large cell-to-cell variability. In such situations inferring a single cell model from cell population data can lead to biologically meaningless results. In order to understand the dynamical behavior of heterogeneous cell populations, it is crucial to develop cell population models, describing the whole population and not only a single individual [1-4].

This has already been realized by several authors, and it has been shown that stochasticity in biochemical reactions and unequal partitioning of cell material at cell division can lead to complex population dynamics [1-5], such as bimodal distributions. Besides these sources for heterogeneity also genetic and epigenetic differences have to be considered [6].

For the purpose of this paper, heterogeneity in populations is modeled by differences in parameter values and initial conditions of the model describing the single cell dynamics [4,7,8]. The network structure is assumed to be identical in all cells. The distribution of the parameter values within the cell population is described by a multi-variate probability density function, which is part of the population model. This modeling framework is well suited for modeling genetic and epigenetic differences among cells [2,4,7].

In the following, the problem of estimating the probability density of the parameters is studied. Therefore, we employ population snapshot data (PSD), which provide single cell measurements at every time instance but which do not provide single cell time series data. A typical experimental setup which provides PSD is flow cytometric analysis. In general, PSD are a common data type in the experimental analysis of biological systems.

So far, there are not many methods available for the estimation of parameter distributions. In pharmacokinetic studies mixed effect models [9] are used frequently. Unfortunately, as in the problem we consider the number of individuals is very large (> 10^4^) and the amount of information per individual very limited (often only one data point), these methods are computationally too demanding. Furthermore, as in this study we are particularly interested in intracellular signal transduction, also methods which purely focus on the population balance [10-12] cannot be employed. In [8,13,14] methods are proposed which can in principle deal with the problem at hand. There, the considered estimation problem has been formulated as a convex optimization problem. Unfortunately, these methods either require an extensive amount of measurement data [8,13], and/or do not allow considering prior knowledge [8,13,14]. Additionally, no methods to evaluate the reliability of the estimates are provided.

In this paper a novel Bayesian approach [15,16] for inferring the parameter density will be introduced. The approach is mainly based on the maximum likelihood methods presented in [13,14], but can deal with sparse and noisy single cell data in addition to realistic measurement noise models. Furthermore, one may directly access the remaining uncertainty of the estimation result and the prediction uncertainties via the calculation of Bayesian confidence intervals [17,18]. It is shown that the posterior distribution can be determined efficiently employing a parameterization of the parameter density in combination with commonly used Markov chain Monte Carlo (MCMC) sampling techniques [19].

To illustrate the properties of the proposed methods, a mathematical model of the tumor necrosis factor (TNF) pathway [20] is analyzed using artificial experimental data.

## Methods

### Problem statement

#### Cell population model

For the purpose of this work we consider intracellular biochemical reaction networks which are modeled by systems of ordinary differential equations. This modeling framework allows to describe metabolic networks as well as signal transduction pathways, as long as spatial effects and stochasticity of the biochemical reactions can be neglected. Mathematically, the dynamic behavior of each single cell is determined by an ordinary differential equation in state space form(1)

with state variables , output variables , and parameters . The vector field  is Lipschitz continuous and the functions  and  are continuous. If for example the concentration  is measured via flow cytometry, we would have , where *c *is a proportionality factor. The index *i *specifies the individual cells within the population. The parameters *θ*^(*i*) ^can be kinetic constants, e.g. reaction rates or binding affinities.

Employing the definition of the single cell dynamics (1), a cell population model is given by the collection of *N *cells,(2)

The heterogeneity within this population is modeled by differences in parameter values among individual cells. The parameters are distributed according to the probability density function , with . This density function is part of the population model specification. The parameter vector of cell *i *is subject to the probability distribution(3)

Note that interactions among individual cells influencing the analyzed pathway are not allowed. This is a restriction but indeed fulfilled in many *in vitro *lab experiments.

#### Measurement data and noise

In this paper we consider experimental setups where measurement data are obtained in the form of population snapshots, e.g. via flow cytometry. Population snapshots are taken at different times *t_j_*, and the *j*th snapshot contains measurements for the output *y *of *M_j _*cells. Due to experiment setup, it can be assumed that any cell is present at most in one snapshot.

The cells in the individual snapshots are referenced through index sets: snapshot *j *contains all cells from the index set , with *M*_0 _= 0. Thereby,  in which  is the number of snapshots.

**Figure 1:**
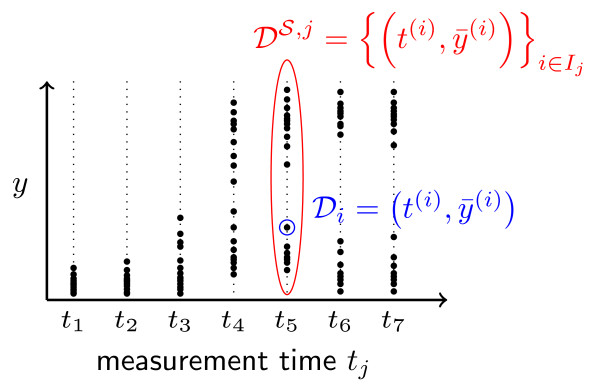
**Population snapshot data of heterogeneous cell population**. The single cell measurement (·) is denoted by  and the snapshot at a particular time instance *t_j _*is denoted by . To the collection of all data we refer as .

Let the data point for the cell with index *i *be denoted as(4)

where *t*^(*i*) ^is the time at which the measurement was taken, and  is the measured, noise-distorted output as defined below. If cell *i *has been measured as part of the *j*th snapshot, then *t*^(*i*) ^= *t_j_*. The snapshot *j *is the set of all data points  with *i *∈ *I_j_*, as depicted in Figure 1:(5)

In the parameter estimation, only the union of all snapshots is considered, and the parameter density function Θ is fitted to all snapshots simultaneously. To this end, we introduce the collection of all data, denoted as(6)

in which  is the total number of measured cells.

We emphasize that experimental setups are considered in which cells are not tracked over time. These setups are very common in studies on the population scale. Classical examples for measurement techniques yielding such data are flow cytometric analysis and cytometric fluorescence microscopy. These measurement techniques allow to determine protein concentrations within single cells. As the population is well mixed when the measurement is performed and no cell is measured more than once, the individual single cell measurements  are independent. This independence of  and  (respectively  and , *i*_1 _≠ *i*_2_, holds if both cells are measured during one snapshot  as well as if the cells are measured within different snapshots .

Like most other experiments also the considered single cell experiments are subject to noise. We consider the noise model(7)

in which  is the measured output and *y*^(*i*) ^is the actual output from (1). The multiplicative noise is denoted by η^× ^∈ ℝ*^m ^*and the additive noise s denoted by η^+ ^∈ ℝ*^m^*. Both, *η*^× ^and *η*^+ ^are in the following assumed to be vectors of log-normally distributed random variables with probability density functions(8)

for all *j *= {×, +}, *k *= 1, ..., *m*. This noise model allows the study of relative and absolute measurement noise and describes the commonly seen noise distributions in biological experiments [21].

**Figure 2:**
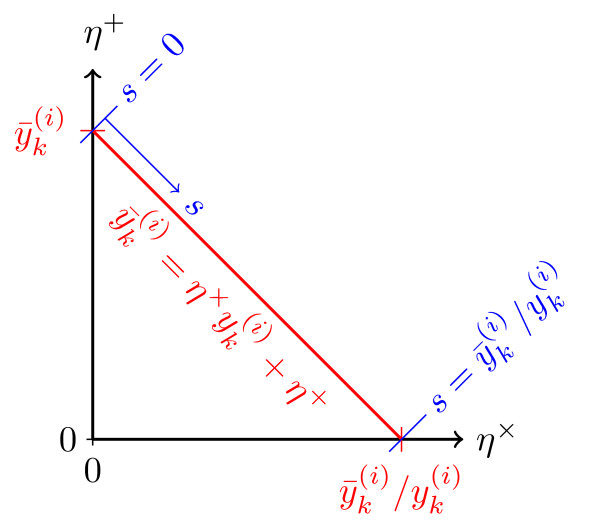
**Line integral (10)**. Set (*red line*) of measurement noise realization *η*^× ^and *η*^+ ^which yield for a given output  the measured, noise corrupted output .

From (8) the conditional probability of measuring  given *y*^(*i*) ^can be determined. As the different output errors are assumed to be independent the conditional probability density is(9)

with  being the value of the line integral(10)

which is illustrated in Figure 2. For this line integral no explicit solution can be given. In this paper its value is determined numerically using the adaptive Simpson quadrature method [22] implemented in MATLAB.

#### Problem formulation

As mentioned previously, when studying heterogeneous populations the density of the parameters Θ is in general unknown but necessary to gain an in-depth understanding of the population dynamics. Therefore, we are concerned with the problems:

**Problem 1 ***Given the measurement data , the cell population model *Σ_pop_, *and the noise model *(8)*, infer the parameter density *Θ*.

**Problem 2 ***Given the measurement data , the cell population model *Σ_pop_, *and the noise model *(8)*, determine the uncertainty of the estimated parameter density *Θ*.

Unfortunately, the number of cells considered in a standard lab experiment is on the order of 10^4 ^to 10^7^. Thus, simulating the population model (2) is computationally expensive. Furthermore, it is hard from a theoretical point of view to deal with ensemble models such as (2). Density-based descriptions of the population dynamics are far more appealing for solving Problem 1 and 2. Therefore, in the next section a density description of the population is introduced.

### Density-based modeling of cell populations

To simplify the inference problems on Θ the population description is changed from an ensemble model (2) to a density model [13]. The variables of this new model are no longer states or outputs of the single cells but the density function ϒ of the output, with . and  This density function yields the probability of drawing a cell sample from the cell population with output ,(11)

in which  is an arbitrary subset of the output space. Hence, *y*^(*i*)^(*t*) can be interpreted as a random variable which is distributed according to ϒ(*y*|*t*,Θ).

**Figure 3:**
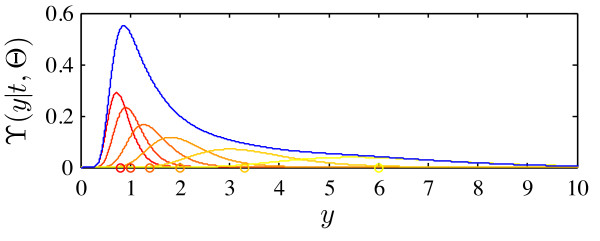
**Illustration of kernel density estimation**. The kernel density estimate (*blue line*) of ϒ(*y*|*t*, Θ) for the measured outputs *y*^(*i*)^(*t*) (*reddish/yellowish circles*) is constructed from the associated kernels  (*reddish/yellowish lines*).

To compute the cell population response ϒ(*y*|*t*,Θ) for a given Θ, *S *independent single cell trajectories *y*^(*i*)^(*t*) of the cell population (2) are calculated. The parameters for these cells are drawn from Θ and the initial conditions are computed according to *x*_0_(*θ*^(*i*)^). This yields  with distribution of *y*^(*i*)^(*t*) depending on Θ. Given  an approximation of ϒ is(12)

in which  is the density of the applied kernel density estimator, with . This is illustrated in Figure 3. In this work multivariate log-normal kernels(13)

are used to conserve the property that all variables are positive. The positive definite matrix *H *is used to select the width of the kernel , and is chosen using the available rule of thumb described in [23]. The selection of *H *is crucial for achieving a good approximation of the probability density, and depends strongly on *S*.

Approaches similar to the one we use to approximate ϒ(*y*|*t*,Θ) are employed in [13,14], in [8] with a naive density estimator and in [24] in the context of cell migration.

### Estimation of the parameter density

In the previous section an approach to determine the output density ϒ within the cell population for a given parameter density Θ is presented. Based on this an approach for estimating Θ from the available data  is developed next.

#### Bayes' theorem for heterogeneous cell populations

For learning the parameter density from the data Bayes' theorem(14)

is used, in which *p*(Θ) is the prior probability of Θ,  is the conditional probability of observing  given Θ,  is the posterior probability of Θ given , and  is the marginal probability of . As the different single cell measurements are independent (14) can be written as(15)

in which  is the conditional probability of observing  given Θ. Note that due to the independence of  and , for *i*_1 _≠ *i*_2_, it is not necessary to distinguish between the cases that (1) the two cells are measured at the same instance  and that (2) the two cells are measured at different time instances . Hence, merely the conditional probability of each individual single cell measurement has to be determined. For the considered process the  can be determined using the output density ϒ,(16)

As this equation cannot be solved explicitly the integral has to be approximated numerically. This could be realized using importance sampling [19], but as drawing a independent sample from ϒ requires knowledge of ϒ in the first place,  is for computational purposes expressed as an integral over *θ*,(17)

in which , with *y*(*t*^(*i*) ^, *θ*) being the output at time *t*^(*i*) ^for a cell having parameters *θ*. This reformulation of (16) is possible as ϒ directly depends on Θ. This step simplifies the evaluation of  tremendously.

Based on (15) and (17), the calculation of the posterior probability for a given probability density of the parameters Θ is possible. Unfortunately, the inference problem nevertheless cannot be solved directly, as Θ is element of a function space, and hence further steps are necessary.

#### Parameterization of parameter density

In order to avoid the infinite dimensional inference problem the parameter density is parameterized. Θ is modeled by a finite weighted sum of multivariate ansatz functions Λ*_j_*,(18)

The ansatz functions  are themselves probability densities with . The weighting vector is denoted by , where *n_φ _*is the number of ansatz functions and  to guarantee that Θ*_φ _*is a probability density. The weightings *φ_j _*can be interpreted as parameters determining the probability density Θ*_φ _*and are for the remainder also called density parameters.

Note that the method presented in the following is independent of the choice of ansatz functions. Nevertheless, a clever choice of the ansatz functions may improve the approximation of the true parameter density dramatically. In this work, the ansatz functions are chosen to be multivariate Gaussians.

Given a parameterization of Θ*_φ_*, the output density can be simplified to

in which ϒ (*y*|*t*, Λ*_j_*) is the output density obtained for single cell parameters distributed according to Λ*_j_*. This representation of the response is possible as the output density fulfills the superposition principle with respect to the parameter distribution Θ*_φ_*. This reformulation has the advantage that computing the output density for arbitrary density parameters *φ *only requires the non-recurring computation of the responses ϒ (*y*|*t*, Λ*_j_*) and summation of those.

#### Reformulation of posterior probability

Having parameterized Θ*_φ _*and ϒ (*y*|*t*, Θ*_j_*) the conditional probability  may be parameterized and expressed as the weighted sum,

in which  is the conditional probability of observing  given the parameter density Λ*_j_*. As the ansatz functions are predefined the conditional probability  can be evaluated,(24)

This in general high-dimensional integral is approximated employing Monte Carlo integration, yielding(25)

in which *θ*^(*k*) ^is drawn from Λ*_j_*, *θ*^(*k*) ^~ Λ*_j_*, and *S_c _*is the total size of the Monte Carlo sample {*θ*^(*k*)^}*_k_*. If Λ*_j _*allows for an efficient drawing of samples, the computational cost of determining  is reasonable, requiring *S_c _*simulations of the single cell model (1).

Given these precomputed 's, which are independent of the density parameters *φ*, the conditional probability can be simplified to(26)

in which  and 〈·,·〉 denotes the scalar product. Employing (26) the posterior probability can be written as(27)

where the prior probability,(28)

enforces the satisfaction of the constraint of Θ*_φ _*being a probability density. Note that for parameter estimation often only the shape of the posteriori probability density is of interest, and not the normalization. Therefore, we only consider(29)

in which  is the unnormalized posterior probability. Sampling from  and  will yield the same distribution of sample members. Furthermore,  and  have the same extrema.

#### Computation of maximum posterior probability estimate

Given the simplified unnormalized posterior probabilities  one important question is which parameter density Θ*_φ _*maximizes . This is the most likely parameter density for the measured data and the prior knowledge.

This optimal parameter density  can be computed by solving the optimization problem(30)

in which the two constraints ensure that the obtained density is positive and has integral one. Note that as Λ*_j _*is a probability density,  is positive if all *φ_j _*are positive. Employing this and optimizing - instead of , (30) can be simplified to(31)

This minimization problem can for concave *p*(Θ*_φ_*) be solved rather efficiently, as in such case (31) is a convex optimization problem [25]. For this problem solvers exist which guarantee convergence to the global optimum in polynomial time, e.g the interior point method [25].

#### Uncertainty of parameter density

In the previous section a method is presented which allows the computation of the maximum posterior probability estimate . As measurement data are limited and noise corrupted this estimate will not be the true parameter density. Hence, the uncertainty of the parameter density has to be evaluated.

##### Sampling of posterior probability density

In order to analyze the uncertainty of the estimate, a sample of the posterior probability density  is generated. This is possible, as the unnormalized posterior probability of a distribution  can be evaluated efficiently given (24) - (28). In this work the sampling is performed with a classical Metropolis-Hastings method [19]. Also Gibbs or slice sampling approaches may be employed. Compared to importance and rejection sampling these methods are well suited as they do not require the selection of an appropriate proposal density, a task which is difficult in this case.

The main point of concern when using MCMC sampling for the problem at hand is that the prior probability and the posterior probability respectively are only non-zero on a (*n*_*φ *_- 1) -dimensional subset of the density parameter space (28). This is due to the fact that the sum over the elements of *φ *has to be one for Θ*_φ _*being a probability density. If a standard proposal step was used, the acceptance rate would have been zero.

This problem can be overcome by performing the sampling in the (*n*_*φ *_- 1)-dimensional space, , and computing the remaining density parameter via the closing condition . According to this the update step for *φ *consists of two steps:

1. Draw proposals  from the (*n*_*φ *_- 1)-dimensional reduced proposal density *T_r_*,(32)

2. Determine  such that ,(33)

In this work, the reduced proposal density is chosen to be a multivariate normal distribution, , with covariance matrix .

This two-step proposal generation procedure is in the following denoted by *φ*^*k*+1^~*T*(*φ*^*k*+1^|*φ*^*k*^). The proposed density parameter vector *φ*^*k*+1 ^is accepted with probability(34)

The distinction of the two cases is hereby crucial to ensure that only probability densities  which are greater than zero for all  are accepted.

By combining update and acceptance step one obtains an algorithm which draws a sample of weighting vectors , or respectively parameter densities , from the posterior distribution. The number of sample members is thereby *S*_*φ*_. The pseudo code for the routine is given in Algorithm 1. In particular, the facts that

• the conditional probabilities  are only computed once in the beginning, and that

• every evaluation of the acceptance probability *p_a _*requires only a small number of algebraic operations,

ensure hereby an efficient sampling. Without this reformulation the integral defining the conditional probability  would have to be computed in each update step. The resulting computational effort would be very large.

**Algorithm 1 **Sampling of posteriori distribution 

   **Require: **data , prior *p*(Θ_*φ*_), model *p*(*y*|*θ *), ansatz functions , initial point *φ*^0^.

      Calculation of conditional probabilities  employing *p*(*y*|*θ *).

      Initialize the Markov Chain with *φ*^0^.

      **for ***k *= 1 to *S*_*φ *_**do**

         Given *φ^i ^*propose *φ*^*k*+1 ^from proposal density *T *(*φ*^*k*+1^|*φ^k^*).

         Calculate posterior probability .

         Generate uniformly distributed random number *r *∈ [0,1].

         **if ***r *<*p_a_*(*φ*^*k*+1^|*φ^k^*) **then**

            Accept proposed parameter vector *φ*^*k*+1^.

         **else**

            Restore previous parameter vector, *φ*^*k*+1 ^= *φ^k^*.

         **end if**

      **end for**

end

##### Bayesian confidence intervals

The sample  generated by Algorithm 1 contains information about the shape of the posterior density . This information can be employed to determine the Bayesian confidence intervals, also called credible intervals.

In this work an approach is presented which employs the percentile method [17] to analyze the uncertainty of Θ*_φ_*. The 100*α*-th percentile of a random variable *r *is the value  below which 100*α *% of the observations fall. Accordingly, the 100(1-*α*)-th percentile interval of *r *is defined as . The Bayesian confidence interval is frequently defined as the 95-th percentile interval [18]. Thus, the parameter is contained in  with a probability of 95% given the measurement data and the prior knowledge.

For the problem of estimating parameter densities, the 100*α*-th percentile is not simply a number but a function. As we are interested in the confidence intervals of Θ*_φ_*(*θ*), the percentiles are defined point-wise for every *θ*. The 100*α*-th percentile of Θ*_φ_*(*θ*) is thus the function  which gives for every parameter vector *θ *the value under which 100*α *% of the observations fall,(35)

Consequently, the 100(1-*α*)% Bayesian confidence interval  of Θ*_φ_*(*θ*) is defined as(36)

As the sample  is given, an approximation of  and  can be obtained by studying the percentiles of the sample [26]. For instance the nearest rank method or linear interpolation between closest ranks can be used to determine .

### Predictions of output density

As the parameter density is not known precisely, also the model predictions show uncertainties. To evaluate the reliability of the population model and its predictive power, these prediction uncertainties have to be quantified. Therefore, the Bayesian confidence interval of the prediction around the output density with the highest a posteriori probability density,(37)

is determined.

The 100(1-*α*)% confidence intervals of the predictions  are again defined via the percentile method,(38)

in which the 100*α*-percentile  of the predicted out put ϒ(*y*|*t*,Θ*_φ_*) is defined as(39)

Computing  for an experiment is a three step procedure. At first, the outputs ϒ(*y*|*t*,Λ*_j_*) (12) are computed. Given ϒ(*y*|*t*,Λ*_j_*) and the sample from the posterior density , a sample from the predicted output density  is given by(40)

This sample can be used to approximate the prediction confidence interval . As the population model has to be simulated only *n_φ _*times, this calculation is computationally tractable.

**Figure 4:**
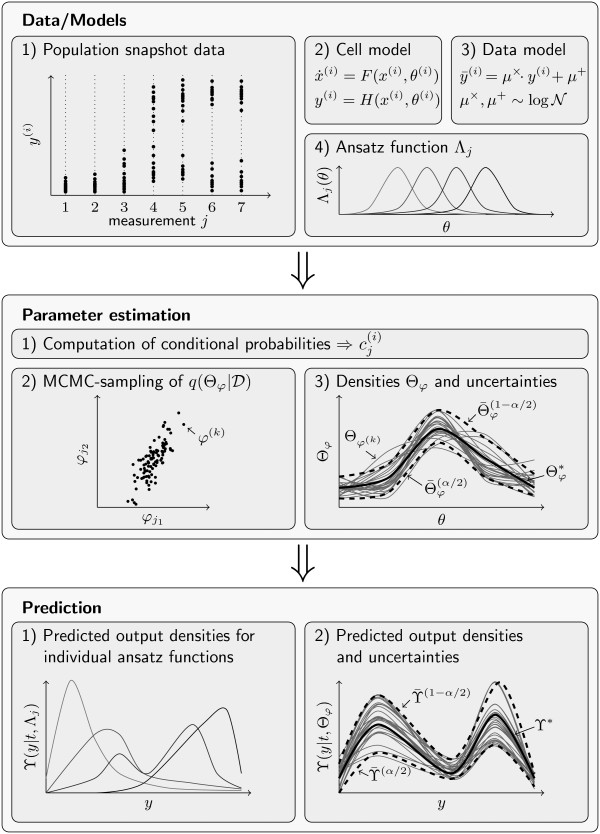
**Illustration of the analysis procedure**. The main steps as well as their order is shown.

To sum up, in this section a method for the estimation of parameter distributions in heterogeneous cell populations from population data has been presented. It has been shown that the optimal value as well as the Bayesian confidence intervals can be computed efficiently employing a parameterization of the parameter density. Also a method to determine prediction uncertainties has been presented. This allows an in-depth analysis of the reliability of the model. A summary of the procedure is shown in Figure 4.

## Results and discussion

To illustrate the properties of the proposed methods, artificial measurement data of a cell population responding to a tumor necrosis factor (TNF) stimulus will be analyzed. For illustration purposes, we consider a case where only one parameter is distributed in a first step. In a second step, we show that the method is also applicable in the case of multi-parametric heterogeneity.

In multicellular organisms, the removal of infected, malfunctioning, or no longer needed cells is an important issue. Therefore, multicellular organisms developed different mechanisms to externally enforce cell death. Thereby the signaling molecule TNF is one of the key players.

TNF can bind to specific death receptors in the cell membrane and is able to induce programmed cell death, also called apoptosis, via the activation of the caspase cascade. On the other hand, it promotes cell survival via the inflammatory response, specifically activation of the NF-*κ*B pathway [27]. The proportion of the activation of these two signaling pathways decides about the fate of the single cell. In the following a simple model for the caspase and NF-*κ*B activation is studied which reproduces the main features of the single cell response to a TNF stimulus.

### Model of TNF signaling pathway

**Figure 5:**
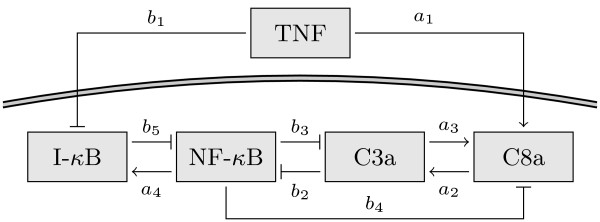
**Graphical representation of the TNF signal transduction model**. Normal arrows indicate activation while arrows with flat hats indicate inhibition.

The model considered in this study has been introduced in [20] and is based on known activating and inhibitory interactions among key signaling proteins of the TNF pathway. A schematic is shown in Figure 5. Besides active caspase 8 (C8a) and active caspase 3 (C3a), the nuclear transcription factor *κ*B (NF-*κ*B) and its inhibitor I-*κ*B are considered in the model. The model is given by the ODE system(41)

The state variables *x_i_*, *i *= 1, ..., 4 denote the relative activities of the signaling proteins C8a, C3a, NF-*κ*B and I-*κ*B, in this order. The functions act*_j_*(*x_i_*) and inh*_j_*(*x_i_*) represent activating and inhibiting interactions, respectively. They are given by(42)

and(43)

The parameters *a_j _*and *b_j _*are activation and inhibition thresholds, respectively, and take values between 0 and 1. The external TNF stimulus is denoted by *u*. Initial conditions of the single cells are the steady states with C3a = 0 for *u *= 0. All nominal parameter values are given in Table 1.

**Table 1:** Nominal parameter values for the TNF signaling model (41).

*i*	1	2	3	4	5
*a_i_*	0.6	0.2	0.2	0.5	
*b_i_*	0.4	0.7	0.3	0.5	0.4

It is known from experiments that the cellular response to a TNF stimulus is highly heterogeneous within a clonal cell population. Some cells die, others survive. The reasons for this heterogeneous behavior are unclear, but of great interest for biological research in TNF signaling, e.g. the use of TNF or related molecules as anti-cancer agent.

To understand the biological process at the physiological and biochemical level it is crucial to consider this cellular heterogeneity, using for example cell population modeling. Here, we model heterogeneity at the cell level via differences in the parameters *b*_3 _and *a*_4_. The parameter *b*_3 _describes the inhibitory effect of NF-*κ*B via the C3a inhibitor XIAP onto the C3 activity, and the parameter *a*_4 _models the activation of I-*κ*B via NF-*κ*B. Studies showed that these two interactions show large cell-to-cell variability [4,7,28].

### Univariate parameter density

For a first evaluation of the proposed method an artificial experimental setup is considered in which the caspase 3 activity is measured at four different time instances during a TNF stimulus,(44)

**Figure 6:**
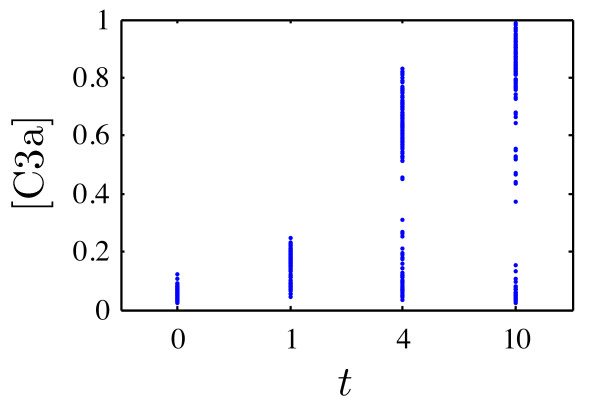
**Artificial population snapshot data of C3a used to infer the parameter density within the cell population**. Each *blue dot *represents a single measured cell.

At each time instance the C3a concentration in 150 cells is determined, *y *= *x*_2_, with measurement noise according to (7), where *μ*^× ^= 0, *σ*^× ^= 0.1, *μ*^+ ^= log(0.05), and *σ*^+ ^= 0.3. This corresponds to an average noise level of about 15%. The generated artificial experimental data for a bimodal distribution in *b*_3 _are depicted in Figure 6.

**Figure 7:**
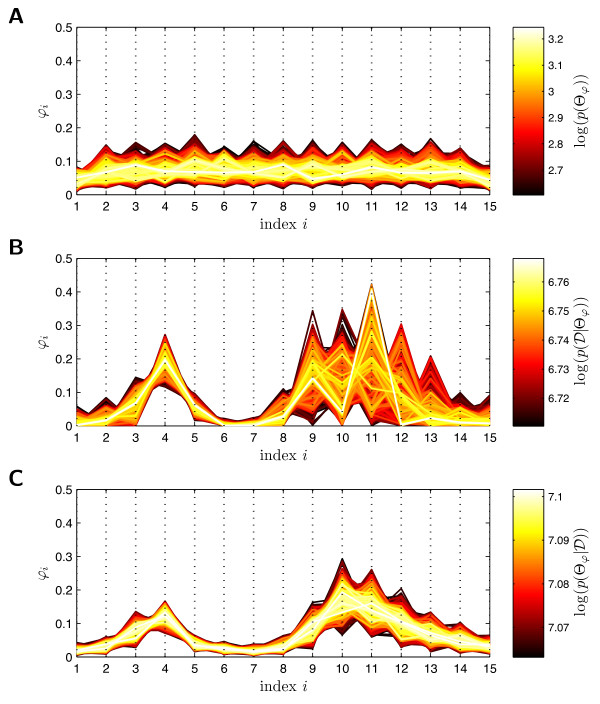
**Visualization of 15-dimensional MCMC sample  from prior, conditional and posterior probability density**. **A**. Plot of sample  drawn from prior probability density of Θ*_φ_*(*b*_3_), *p*(Θ*_φ_*). **B**. Plot of sample  drawn from conditional probability density of Θ*_φ_*(*b*_3_), . **C**. Plot of sample  drawn from posterior probability density of Θ*_φ_*(*b*_3_), . Each polyline represents hereby one point *φ^k ^*in the 15-dimensional density parameter space. The position of the vertex on the *i*-th dotted vertical line gives the value of the *i*-th density parameter. The color of the points indicates the logarithm of the unnormalized probability density of the data. Bright polylines (points *φ^k^*) have a high posterior probability whereas dark polylines have a low posterior probability.

**Figure 8:**
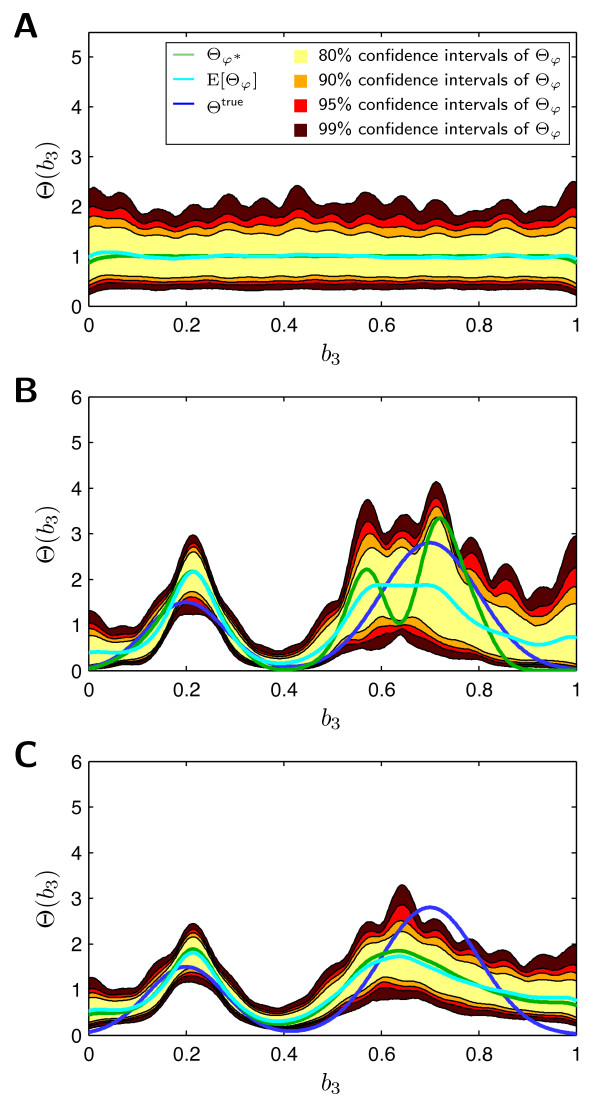
**Prior, conditional and posterior probability density of Θ*_φ _*(*b*_3_) in *b*_3 _- Θ(*b*_3_) - plane**. **A**. Plot of prior probability density of Θ*_φ_*(*b*_3_), *p*(Θ*_φ_*). **B**. Plot of conditional probability density of Θ*_φ_*(*b*_3_), . **C**. Plot of posterior probability density of Θ*_φ_*(*b*_3_), . The colored lines indicate the distribution with the highest posterior probability , and the mean distribution E[Θ*_φ_*], for the individual probability densities, as well as the true parameter distribution Θ^true^. The colored regions indicate the 80%, 90%, 95%, and 99% Bayesian confidence intervals of the parameter distribution Θ*_φ_*.

As ansatz functions Λ*_j _*for the estimation, we use *n_φ _*= 15 truncated Gaussians(45)

where  = and *s_j _*such that . The center points *μ_j _*are equidistantly distributed on the interval 0[1]. The prior probability *p*(Θ*_φ_*) is chosen to be(46)

in which *p_β _*is the probability density of the beta-distribution. The parameter *α_j _*and *β_j _*are selected such that *p_β _*(*φ_j_|α_j_|β_j_*) has its extremum at  and convariance *σ*^2^. The distribution of a sample {*φ^k^*}*_k _*drawn from this prior is shown in Figures 7 and 8. Note that the prior does not enforce a trend to smaller or larger parameter values of *b*_3_. Furthermore, it does not enforce a trend to unimodal or bimodal distributions Θ*_φ _*(*b*_3_). Such distribution properties shall be inferred from the data.

Given the ansatz functions Λ*_j _*(45) the conditional probabilities  of observing  are determined using importance sampling, according to (25). This computation takes about three minutes, on a standard personal computer using a single CPU. Thereby, 32% of the computation time are required for the simulation of the individual cells *y*(*t*, *θ*^(*j*)^) for individual parameter values *θ*^(*j*)^, and 59% for the evaluation of the conditional probability . The rest is spent on pre- and post-processing. Subsequently, MCMC sampling is performed to obtain a sample  of the prior (with *σ*^2 ^= 0.05), of the conditional, and of the posterior probability distribution. The sample has *S_φ _*= 10^6 ^members and the generation takes only four minutes. The computation is very fast, as the proposed approach simplified the evaluation of the conditional probability to a matrix vector multiplication. Note, that all steps during the computation of the conditional probabilities and the MCMC sampling can be parallelized, yielding a tremendous speed-up for more complex models.

The results of the sampling are illustrated in Figure 7 using parallel coordinates [29]. From this representation of  it can be seen that after the learning processes most of the density parameters still show large uncertainties. The uncertainty in the posterior distribution is a lot smaller than the uncertainty in the likelihood function, due to the stabilization via the prior. Note that the visualization also uncovers pronounced correlations between some parameters, e.g. *φ*_10 _and *φ*_11 _are negatively correlated for . This indicates that the model of the density of *b*_3 _is over-parameterized with respect to the data. Thus, the number of ansatz functions could be reduced while still achieving a good fit.

To analyze the uncertainty of Θ*_φ _*in more detail the sample  is employed to determine the 80%, 90%, 95%, and 99% Bayesian confidence intervals. The results are depicted in Figure 8. It can be seen that the confidence intervals for some values of *b*_3 _are rather small, indicating that the data contain many information about these regions. Unfortunately, in particular for *b*_3 _> 0.6 the confidence intervals are very wide showing that the parameter density in this area cannot be inferred precisely. But, although the amount of data is limited and the uncertainty with single *φ_i_*'s may be large, the posterior distribution of Θ*_φ _*already shows key properties of the true parameter density, e.g. the bimodal shape, which has not been provided as prior information. This bimodal shape is also seen in the likelihood function, but there the uncertainties are larger than in the posterior probability distribution.

**Figure 9:**
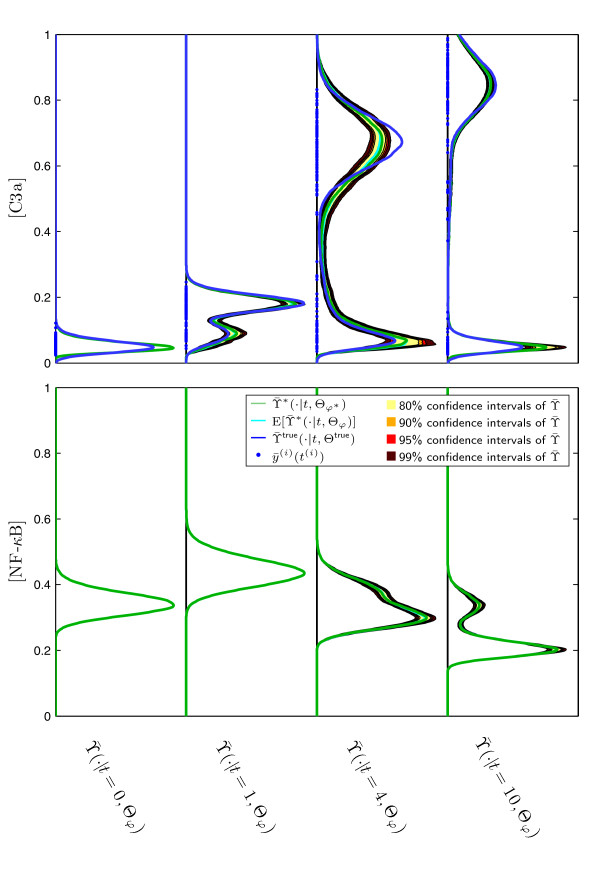
**Predicted measured output densities  and **. The colored lines indicate the distribution with the highest posterior probability , and the mean distribution , as well as the true measured output distribution . The colored regions indicate the 80%, 90%, 95%, and 99% Bayesian confidence intervals of the predicted distribution .

Besides the uncertainty of Θ*_φ _*also the predictive power of the model can be evaluated. This is exemplified by studying the confidence interval of  and  for the previously considered experimental setup. The bar indicates that the distribution of the noise corrupted output  instead of the true output *y *is considered. This allows the direct comparison of the prediction with the data. The predictions are shown in Figure 9.

It is obvious that, although the parameter distributions show large uncertainties, the predictions are rather certain. This is indicated by tight confidence intervals. Furthermore, the mean predictions  and the predictions with highest posterior probability  agree well with the true output distribution , for measured output C3a and predicted output NF-*κ*B. The small prediction uncertainties can be explained to be sloppiness [30] of the density parameters *φ_i _*parametrizing the distribution of *b*_3_. A detailed analysis indicates (not shown here) that the number of ansatz function can be decreased, still ensuring a good approximation of the distribution of *b*_3_.

### Multivariate parameter density

In many biological systems several cellular parameters are heterogeneous and different cellular concentrations can be measured [7]. Therefore, we show in this section that the proposed method can also be employed to estimated multivariate parameter densities from multi-dimensional outputs. Furthermore, the influence of the choice of the prior on the estimation result is analyzed.

**Figure 10:**
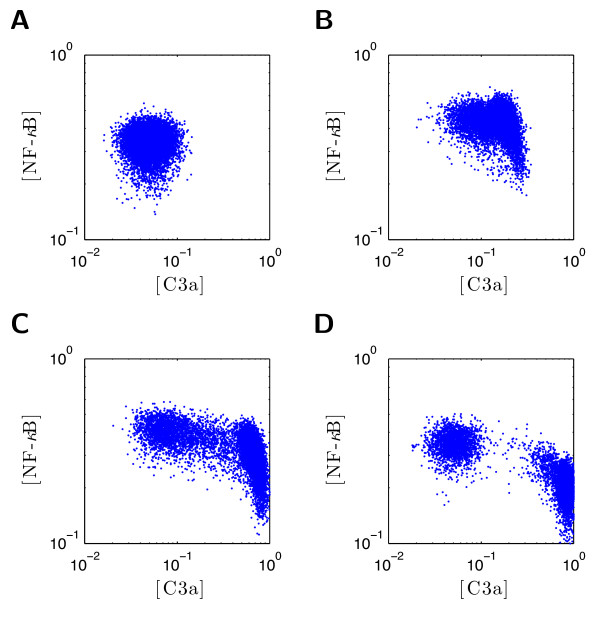
**Artificial population snapshot data of C3a and NF-*κ*B used to infer the parameter density within the cell population**. **A**. Plot of snapshot data for *t *= 0. **B**. Plot of snapshot data for *t *= 1. **C**. Plot of snapshot data for *t *= 4. **D**. Plot of snapshot data for *t *= 10. Each *blue dot *represents a single measured cell.

To perform this study we considered the same experimental setup as above. The only difference is that two concentrations are measured, C3a and NF-*κ*B, *y *= [*x*_2_, *x*_3_]*^T^*. The considered artificial experimental data of 10^4 ^cells are depicted in Figure 10. The ansatz function for Θ*_φ _*are *n_φ _*= 100 truncated multivariate Gaussians equivalently to (45). The covariance matrix is 0.06^2 ^· I_2 _and the extrema are equidistantly distributed on a regular 2-dimensional grid which is aligned with the axes.

**Figure 11:**
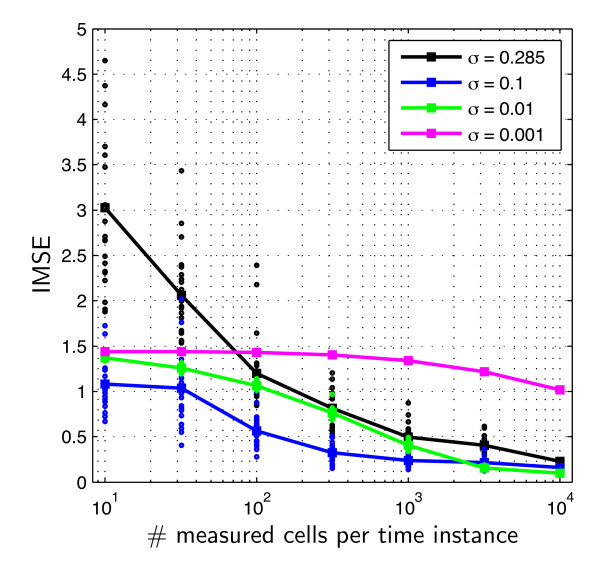
**Integrated mean square error as function of the amount of available data and the informativeness of the prior**. The plot shows the integrated mean square error for different numbers of measured cells per time instance and different standard deviation, *σ*, of the prior. Individual realization (*dots*) and resulting mean (*squares*).

Given this setup, the convergence rate is studied in terms of the integrated mean square error,(47)

of true distribution and distribution with highest posterior probability . The IMSE is computed for amounts of measured cells per time instance and different priors. The priors are thereby again beta-distributions (46). The extrema *φ*_ext _are chosen as in the last section such that the prior is flat. The standard deviation on the other hand is reduced step-wise from *σ *= 0.285 (completely uninformative as almost uniform on the feasible interval  to *σ *= 0.001 (very informative). Given this requirements, the values *α_i _*and *β_i _*of the prior (46) are determined. The result for different numbers of measured cells sampled from the available data set is shown in Figure 11. Note that the IMSE is a stochastic quantity as the selection of measured cells is a stochastic processes and hence also the estimated density  is stochastic. To account for this stochasticity, several realizations are performed and the mean is computed.

**Figure 12:**
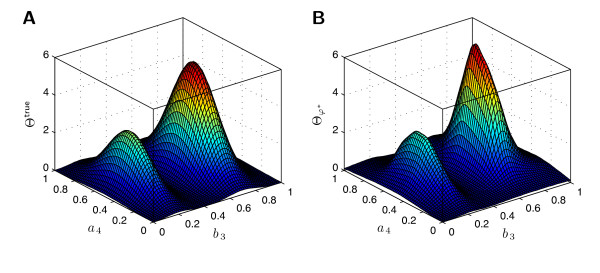
**Estimation result for 2-dimensional parameter density**. **A**. Plot of the true parameter density, Θ^true^. **B**. Plot of the estimated parameter density, . The estimated parameter density is obtained using 10^4 ^measured cells at each time instance and a prior with *σ *= 0.01.

From Figure 11 it becomes clear that the IMSE strongly depends on both, amount of data and informativeness of prior. For uninformative priors, the outcome for little data is highly uncertain and the IMSE is large and shows large variations. On the contrary, if the prior is very informative but wrong, the number of measurement data required to obtain a good estimate is tremendous. For the right choice of *σ*, one observes a fast convergence of the  to Θ^true^, as shown in Figure 12, and only little variation for small amounts of data. Hence, the usage of prior knowledge, even if it is only partially correct, yields for more stable estimates and faster convergence. Furthermore, this study suggests that the typical number of cells measured by flow cytometry (10^4^) is informative enough to infer key features of population heterogeneity.

## Conclusions

In this paper a Bayesian approach for inferring the parameter density for heterogenous cell populations with single cell resolution from population data is presented. We show that the proposed model can deal with limited and noisy measurement data as well as realistic noise models. The method utilizes a parameterization of the parameter density which, in combination with a reformulation of the conditional probability, allows a computationally efficient evaluation of the posterior probability. Compared to other methods for cell populations this approach does not rely on the approximation of the measured population response using density estimators.

For sampling from the posterior probability the Metropolis-Hastings algorithm is used. Here it has been adapted to be applicable to the considered constraint problem. Using this sampling strategy a sample from the posterior probability density is determined. This sample is employed to compute Bayesian confidence intervals for the parameter distribution, as well as for the model predictions. Also summary statistics like mean parameter density and mean predicted output density can easily be determined. For concave prior distributions we could even show that the calculation of the parameter density for the highest posterior probability is a convex problem.

The properties of the proposed scheme are evaluated using artificial data of a TNF signal transduction model. It could be shown that the proposed method yields good estimation results for a realistic experimental setup. Furthermore, although the remaining uncertainties are large, the predictions showg only small uncertainty indicating sloppiness of parameters.

Concerning the choice of the prior distribution it could be shown that the regularizing effect is beneficial if only little data is available. On the other hand, if the amount of available data increases, informative but not carefully chosen priors slow down the convergence.

## Authors' contributions

JH, SW, and PS developed the problem formulation. JH developed the methods and implemented the algorithms. JH, SW, NR, and FA contributed to the systems dynamics and statistics. JH, SW, and FA constructed the application example and JH applied the proposed method. MD and PS contributed to the selection of the studied biological system, the choice of the addressed biological questions, and the interpretation of the results. JH, SW, and NR wrote the article. All authors read and approved the final manuscript.
